# The Protective Effect of Beraprost Sodium on Diabetic Cardiomyopathy through the Inhibition of the p38 MAPK Signaling Pathway in High-Fat-Induced SD Rats

**DOI:** 10.1155/2014/901437

**Published:** 2014-11-11

**Authors:** Jie Li, Li Peng, Hong Du, Yangtian Wang, Bin Lu, Yixin Xu, Xiaozhen Ye, Jiaqing Shao

**Affiliations:** Department of Endocrinology, Jinling Hospital, Medical School of Nanjing University, 305 East Zhongshan Road, Nanjing, Jiangsu 210002, China

## Abstract

*Objective*. To investigate the effect of beraprost sodium (BPS) on diabetic cardiomyopathy and the underlying mechanism. *Methods*. A total of 40 Sprague Dawley rats were randomly divided into the normal control group (*N* = 10) and the model group (*N* = 30). The model group was fed a high-fat diet followed by a one-time dose of streptozotocin (STZ) to establish the diabetes mellitus model. After that, rats were randomly divided into two groups with or without BPS intervention. After 8 weeks, we explored the role of the p38 MAPK signaling pathway in inflammation, oxidative stress, cardiac morphology, and myocardial apoptosis. *Results*. Compared with control, the ratio of heart-weight to body-weight and the serum levels of SOD and GSH in the BPS group significantly increased, the expression of p38 MAPK, the serum levels of MDA, TGF-*β*1, TNF-*α*, HIF-1*α*, MMP-9, caspase-3, BNP, ANP, and heart Bax expression significantly decreased, and heart Bcl-2 expression significantly increased. H&E staining in diabetic rats showed the cardiac muscle fibers derangement, the widening gap, the pyknotic and fragmented nuclei, and more apoptosis. *Conclusions*. BPS effectively showed protective effects on diabetic myocardial cells, possibly through the inhibition of p38 MAPK signaling pathway.

## 1. Introduction

Epidemiological studies indicate that more than 70% of diabetes mellitus (DM) patients die of cardiovascular disease (CVD); this number is 2-3 times higher than the mortality of CVD in the nondiabetic population [1]. The prevention of cardiovascular events is a very important goal in the treatment of DM. Diabetic cardiomyopathy (DCM) is one of the major cardiac complications in DM patients. The incidence of DCM is very high, and the disease is highly dangerous, directly causing mortality due to cardiovascular events in DM patients. In recent years, many large-scale studies confirmed that a reduction in hemoglobin A1C (HbA1C) alone did not benefit the primary cardiovascular endpoint. Therefore, in addition to the control of blood sugar, studying and developing new types of antidiabetic drugs for cardiovascular protection have become popular in the field of DM research.

Mitogen-activatedproteinkinases (MAPKs) are a group of intracellular serine/threonine protein kinases. The MAPK signaling pathway is present in most cells; this pathway transduces extracellular signals into cells and nuclei and plays an important role in biological functions (such as proliferation, differentiation, transformation, and apoptosis). A series of studies in recent years showed that pathological signals such as high glucose, activation of the polyol pathway, and oxidative stress all activated MAPKs; therefore, MAPKs become a converging point for different signaling pathways induced by high glucose. Some scholars have thus regarded MAPKs as signal transducers of DM complications induced by high glucose levels [2]. The activation of the MAPK signal transduction pathways also causes and accelerates the development and progression of DCM to some extent. The MAPK family is involved in a series of changes associated with coronary artery disease such as fibrosis, cell hypertrophy, and migration and is considered to be the primary cause of restenosis after arterial and venous reconstructions [3].

The p38 MAPK signaling pathway is an important member of this family. As a kinase activated by oxidative stress, p38 MAPK primarily participates in apoptosis, immune regulation, cell transdifferentiation, and inflammatory response in response to oxidative stress. The p38 MAPK signaling pathway is activated by many stimulating factors such as reactive oxygen species, inflammatory factors, high glucose, and angiotensin II, thus exacerbating myocardial fibrosis and ischemia [4–6]. Studies showed that inflammation plays an important role in the onset and development of DCM. The p38 MAPK signaling pathway and inflammation may be an important pathogenic mechanism underlying DCM. Studies showed that phosphorylated p38 MAPK (p-p38 MAPK) activated related inflammatory factors such as NF-*κ*B and TNF-*α* and caused a series of proinflammatory responses, thus causing pathophysiological changes such as apoptosis and left ventricular remodeling [7–10]. Pathophysiological mechanisms associated with the development of type-2 DM such as high-glucose toxicity, oxidative stress, and angiotensin II are also indirectly regulated by the p38 MAPK signaling pathway [11]. In cultured endothelial cells, increasing concentrations of glucose further activate the p38 MAPK signaling pathway [12]. These results confirmed that the p38 MAPK signaling pathway plays an important role in the pathogenesis of DCM. The abnormal production of inflammatory factors and chemokines and the differential activation of the p38 MAPK signaling pathway in different cells may be a potential mechanism of pathogenesis underlying the damage of endothelial cells and cardiac function in DM [13].

Prostacyclin (PGI), which was first discovered in 1974, is primarily produced by vascular endothelial cells. PGI is a metabolic product of arachidonic acid and has strong antiplatelet and vasodilation functions. However PGI is also very unstable, has a short half-life, and has a poor oral bioavailability. As a prostacyclin analog (PGI2), beraprost sodium (BPS) avoids the above shortcomings. BPS is the first orally administered prodrug of PGI. Functions of BPS include vasodilation, antiplatelet effects, inhibition of vascular cell adhesion molecule 1 (VCAM-1) expression, inhibition of inflammatory factor release, and inhibition of vascular endothelial injury caused by reactive oxygen species. For these reasons, BPS may be highly effective for the prevention and treatment of microvascular complications of DM. Our previous studies preliminarily showed that BPS treatment effectively reduced the levels of inflammatory factors such as IL-6, myeloperoxidase (MPO), and high-sensitivity CRP (hs-CRP) in rats; improved oxidative stress system disorders in rats; and reduced oxidative stress reactions and inflammatory injury [14]. These benefits were all independent of the reduction of blood pressure; however, whether these benefits have protective effects on DCM remains clear.

Inflammation, oxidative stress, and proliferation of vascular intimal and fibrous tissues are involved in the onset and development of DCM; thus, inflammatory injury and vascular intimal proliferation may become a new therapeutic target for DCM.

Preliminary pharmacological studies showed that p38 MAPK-specific inhibitors had therapeutic effects on myocardial ischemia, myocardial apoptosis, and left ventricular hypertrophy [3]; however, the specific mechanism remains to be elucidated. This study aimed to study the effects of treatment with BPS on type-2 DM rats and to investigate its effect on the p38 MAPK signaling pathway in the heart of type-2 DM rats, its effects on oxidative stress and inflammatory reactions, and its function as a quantitative indicator for myocardial apoptosis and heart failure, which will provide new ideas about the clinical treatment of DCM.

## 2. Materials and Methods

### 2.1. Animals

A total of 40 6-week-old, male, specific-pathogen-free (SPF)-grade Sprague-Dawley (SD) rats with body weights of 180 ± 20 g were purchased from the Animal Center of The Second Military Medical University. Animals were housed in clean-grade animal rooms in the Animal Center of The Second Military Medical University. The animal production permission was SCXK (Shanghai) 2007-0003, and the animal use permission was SYXK (Shanghai) 2007-0003. Animals were housed in different cages with 5 animals in each cage with an artificial light cycle. The light and dark times per day were 12 h/12 h, the temperature was 21 ± 2°C, and the humidity was 55 ± 2%. Animals accessed water ad libitum, and food was adequately provided. Animal treatments were performed in accordance with the principles of experimental animal care (NIH Publication NO85-23, amended in 1985).

### 2.2. Major Reagents and Instruments

Streptozotocin (STZ) was purchased from Sigma. BPS (Dena) was a gift from Astellas Pharma Inc.; DEPC was provided by Shanghai Biocolor BioScience & Technology. Trizol, the RT-PCR reagent kit, and DNA markers were from Invitrogen. Primers for p38 MAPK target genes and the internal control GAPDH were synthesized by Shanghai DaWeiKe Biotechnology. The RNA Guard reagent was purchased from Shanghai Huashun Biological Reagent Co.; the ReverTra Ace reverse transcription reagent kit and SYBR Green Real-time PCR Master Mix were from TOYOBO (Japan). The total cellular protein extraction reagent was from KeyGEN Biotech (Nanjing). The mouse anti-human p-p38 antibody, mouse anti-human total p38 (t-p38) antibody, goat anti-human TNF-*α* antibody, goat anti-human MMP-9 antibody, goat anti-rat COX-2 antibody, goat anti-rat FN antibody, and goat anti-human CREB antibody were from Santa Cruz Biotechnology (USA). TGF-*β*1, SOD, GSH, and MDA assay kits were purchased from Nanjing Jiancheng Biotech (Nanjing, China). Citric acid and sodium citrate were purchased from Sinopharm Chemical Reagent Co., Ltd. and 2.5% glutaraldehyde was purchased for scanning electron microscopy from Fudan University's School of Medicine. 4% paraformaldehyde was from Department of Pathology, Changzheng Hospital. 10% chloral hydrate was from Second Military Medical University Experimental Animal Center. The H110-type analytic balance was from Sartorius (Germany). The ABI7500fast PCR machine was from Applied Biosystems (USA). The 8000B tabletop centrifuge was from Beijing Scientific Instrument Factory, and the UP400S tissue homogenizer was from Shanghai Scientific Instrument Factory. The UP400S sonicator was from Sanyo (USA). The electrophoresis apparatus and the electrotransfer apparatus were from Bio-Rad (USA). The microplate reader was from Bio-Tek (UK). The high-speed low-temperature centrifuge was from Beckman (USA). The magnetic stirring apparatus was from Huamei Biochemistry (Taicang City). The decolorization shaker was from Xinghua Chemical Instrument Factory. The Leica thermostat water bath, Leica CMl900 frozen cut tablet machine, Leica EGl160 paraffin-embedded machine, Leica RM2135 Microtome, Leica DCS00 fluorescence microscope, and Leica image analysis system were from Leica (Germany). Optical microscope was from Olympus (Japan). The incubator was from Taicang Science and Educational Instrument Factory (Jiangsu).

### 2.3. Experimental Grouping

After 1 week of adaptive feeding, SD rats were randomly divided into the control (CN) group (*N* = 10) and the model group based on the random number table method.

*Induction of the DM Rat Model*. The DM model group was fed a high-fat diet for 4 weeks. After the insulin-resistant model was established, STZ (dissolved in 0.1 mol/L citric acid buffer, pH 4.3) was intraperitoneally injected at 30 mg/kg. Two weeks later, rats were fasted for 8 h, and 20% D-glucose solution was administered at 2 g/kg to perform oral glucose tolerance tests. When glucose levels at 0 min and 120 min were higher than 7.0 mmol/L and 11.0 mmol/L, respectively, the type-2 DM rat model was established successfully. There were 24 rats in the successful DM model. The CN group was intraperitoneally injected with the same dose of citric acid buffer (pH 4.3, 0.1 mol/L).

*Animal Grouping and Treatment*. After the model was successfully established, rats were randomly divided into the nonintervention DM group (*N* = 12) and the BPS intervention DM group (DM + BPS; *N* = 12) based on the random number table method. Rats in the DM + BPS group were treated with BPS (0.6 mg/kg/d), whereas rats in the CN and DM groups were intragastrically administered an equal volume of double distilled water at the same time every day. The experiment lasted for 12 weeks (week 0–week 12). The body weight and blood glucose levels of rats were monitored every week. Animals were sacrificed at the end of week 12. At the end of the experiment, 6 animals were included in each experimental group.

### 2.4. Collection of Tissue Samples

After fasting for 12 h, intraperitoneal glucose tolerance tests (IPGTTs) were performed on rats that were about to be sacrificed. Rats were then anesthetized by intraperitoneal injection of 10% chloral hydrate at 4 mg/kg. The abdomen was opened, and blood samples were collected from the abdominal aorta. Samples were placed in tubes with EDTA anticoagulant and centrifuged. Plasma samples were collected and stored in a −20°C freezer for subsequent usage. The heart was rapidly removed, weighed, and stored in liquid nitrogen. Half of tissue samples were fixed in 10% neutral-buffered formalin for histopathological examination. Oxidative stress products, superoxide dismutase (SOD), malondialdehyde (MDA), glutathione (GSH), and other related factors were measured, using blood samples. The activation of the p38 MAPK signaling pathway in myocardial tissues was determined using RT-PCR and Western blotting. In addition, protein levels of TNF-*α*, caspase-3, Bax, Bcl-2, hypoxia inducible factor 1*α* subunit (HIF-1*α*), brain natriuretic peptide (BNP), atrial natriuretic peptide (ANP), and matrix metalloproteinase-9 (MMP-9) were determined.

### 2.5. Oxidative Stress and Fibrosis-Related Factors Were Measured

Blood samples of rats were centrifuged at 3,000 rpm for 15 min at 4°C, using an automatic biochemistry analyzer (Hitachi. 7020, Japan). Malondialdehyde (MDA), glutathione (GSH), and superoxide dismutase (SOD) and transforming growth factor *β*1 (TGF-*β*1) in blood were, respectively, assessed by means of thiobarbituric acid (TBA), chemical colorimetry, xanthine oxidase, and ELISA double antibody sandwich assay.

### 2.6. Detection of p38 MAPK Gene Expression Using RT-PCR (Table 1)

Primers were synthesized by Shanghai DaWeiKe Biotechnology.

Each sample was repeated in 6 wells, and a negative control without template cDNA was also used. Amplification was conducted in an ABI Prism7500 fluorescence quantitative PCR machine. The amplification conditions were 50°C for 2 min; 95°C for 10 min; 40 cycles of 95°C for 15 s; and 60°C for 1 min. After amplification, the melting curve was plotted starting from 60°C to validate the specificity of the amplification products. After the reaction was performed, baseline and thresholds were established, and the threshold cycle (Ct) values were obtained.

### 2.7. Detection of p38 MAPK Signaling Pathway Activation in Myocardial Tissues and the Protein Levels of TNF-*α*, Bax, Bcl-2, HIF-1*α*, BNP, ANP, and MMP-9 Using Western Blotting

One hundred micrograms of rat myocardia was weighed on ice and stored at −80°C after being aliquoted. Protein samples were quantitated using the BCA method; the absorbance of each well was determined using a microplate reader at a wavelength of 560 nm, and a standard curve was plotted. An 8% resolving gel with a 4% stacking gel was prepared, and samples were loaded for electrophoresis. After protein samples were transferred onto a membrane and blocked for approximately 1 h, 1 : 1000 dilutions of the primary antibodies, t-p38 MAPK, p-p38 MAPK, TNF-*α*, TGF-*β*1, Bax, Bcl-2, HIF-1a, BNP, ANP, or MMP-9, were added and incubated at 4°C overnight. Horseradish peroxidase- (HRP-) conjugated secondary antibodies at a 1 : 2000 dilution were added and incubated at 37°C for 1.5 h. After washing with TBST four times for 10 min, protein bands were analyzed using the Bio Image System (a gel documentation system; SYNGENE, A DIVISION of SYNOPTIC, LTD) to obtain optical density values.

### 2.8. Myocardial Paraffin Slicing and Staining with H&E

After sacrificing the rats in each group, tissue blocks from left ventricle were taken, fixed with 4% neutral formalin, dehydrated with graded ethanol, embedded in paraffin, and conventionally prepared into myocardial paraffin slices with a slice thickness of 5 *μ*m and then stained with H&E, and the slices were sealed with neutral gum. Myocardial pathological changes were observed under light microscopy after HE staining.

### 2.9. Calculating the Cardiac Myocyte Apoptotic Index (AI)

Apoptosis was detected by performing the TUNEL assay. Myocytes were considered to be TUNEL-positive when the nuclei were identified as staining dark brown. In each tissue specimen, five high-power fields (×400) were randomly selected; the apoptotic index (AI) was calculated in these fields as the percentage of positive cells, given by the following equation: AI = (number of positive cells/total number of cells) × 100%.

### 2.10. Statistical Analysis

Statistical analyses were performed using the SPSS for Windows 13.0 software. Count data are presented as ($\bar{x}\pm \text{s}$). The comparison of mean values among multiple groups was performed using the one-way analysis of variance (ANOVA); the comparison between two groups was examined using the least significant difference (LSD) test. The comparison of physiological and metabolic indicators before and after drug administration was performed using the paired *t* test. *P* < 0.05 indicated that the difference was statistically significant.

## 3. Results

### 3.1. Ratio of Heart-Weight to Body-Weight

**Figure 1:**
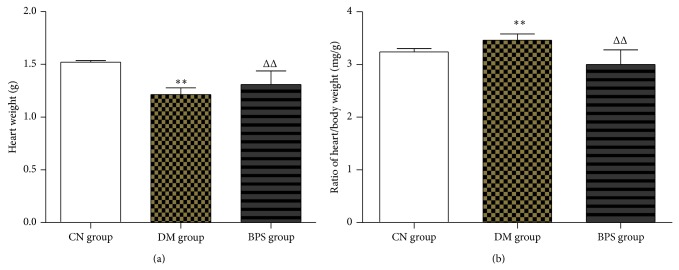
Heart weight and the ratio of heart-weight to body-weight in each group. Compared with the CN group, heart weight significantly decreased in the DM group (*P* < 0.01). Compared with DM group, heart weight increased in the BPS group (*P* < 0.01). Compared with CN group, the ratio of heart-weight to body-weight increased in both DM group and BPS group (*P* < 0.01). Compared with DM group, the ratio of heart-weight to body-weight decreased (*P* < 0.01) in BPS group. There was no statistical difference between BPS group and CN group about the ratio of heart-weight to body-weight.

Compared with the CN group, heart weight significantly decreased in the DM group (*P* < 0.01). Compared with DM group, heart weight significantly increased in the BPS group (*P* < 0.01). Compared with CN group, the ratio of heart-weight to body-weight increased in both DM group and BPS group (*P* < 0.01). Compared with DM group, the ratio of heart-weight to body-weight decreased (*P* < 0.01) in BPS group. There was no statistical difference between BPS group and CN group about the ratio of heart-weight to body-weight (Figure 1).

### 3.2. Oxidative Stress and Fibrosis Factors

**Figure 2:**
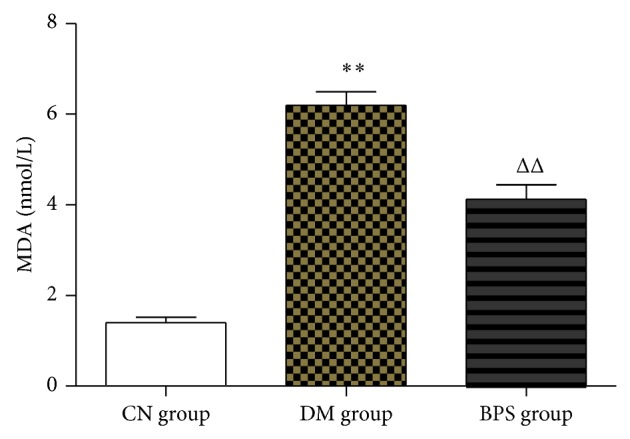
MDA in each group. Compared with CN group, the level of MDA significantly increased in DM group (*P* < 0.01); the level of MDA increased in BPS group, but the difference was not statistically significant (*P* > 0.05). Compared with DM group, the level of MDA significantly decreased in BPS group (*P* < 0.01).

Compared with CN group, the level of MDA significantly increased in DM group, and the difference was statistically significant (*P* < 0.01); the level of MDA increased in BPS group, but the difference was not statistically significant (*P* > 0.05). Compared with DM group, the level of MDA decreased in BPS group; the difference was statistically significant (*P* < 0.01) (Figure 2).

**Figure 3:**
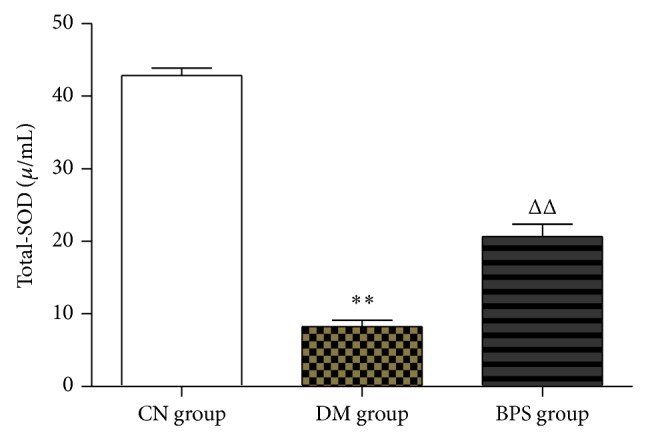
SOD in each group. Compared with CN group, the level of total SOD significantly decreased both in BPS and in DM group (*P* < 0.01). Compared with DM group, the level of total SOD significantly increased in BPS group (*P* < 0.05).

Compared with CN group, the level of total SOD significantly decreased both in BPS and DM group; the difference was statistically significant (*P* < 0.01). Compared with DM group, the level of total SOD significantly increased in BPS group (*P* < 0.05) (Figure 3).

**Figure 4:**
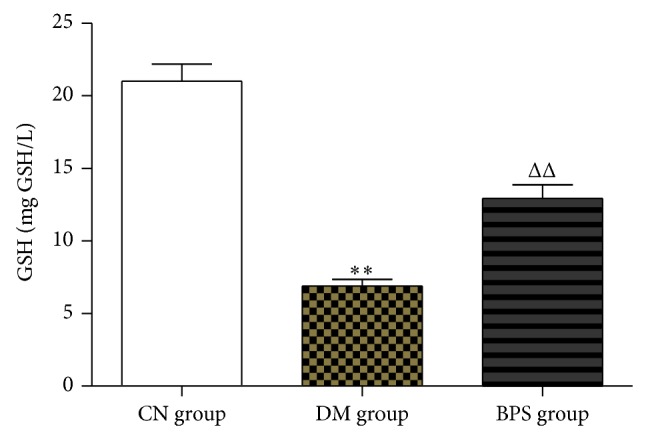
GSH in each group. Compared with CN group, the level of GSH significantly decreased in DM group; the difference was statistically significant (*P* < 0.01). Compared with DM group, the level of GSH increased in BPS group; the difference was statistically significant (*P* < 0.05).

Compared with CN group, the level of GSH significantly decreased in DM group; the difference was statistically significant (*P* < 0.01). Compared with DM group, the level of GSH increased in BPS group; the difference was statistically significant (*P* < 0.05) (Figure 4).

**Figure 5:**
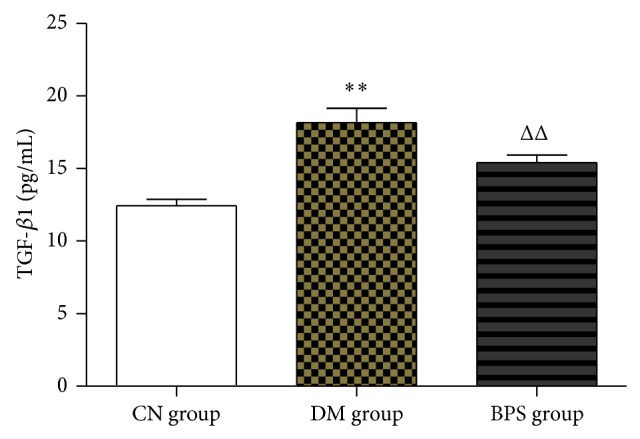
TGF-*β*1 in each group. Compared with CN group, the level of TGF-*β*1 significantly increased in both BPS group and DM group; the difference was statistically significant (*P* < 0.01). Compared to DM group, the level of TGF-*β*1 significantly decreased in BPS group (*P* < 0.01).

Compared with CN group, the level of TGF-*β*1 significantly increased in both BPS group and DM group; the difference was statistically significant (*P* < 0.01). Compared to DM group, the level of TGF-*β*1 significantly decreased in BPS group (*P* < 0.01) (Figure 5).

### 3.3. RT-PCR Results

**Figure 6:**
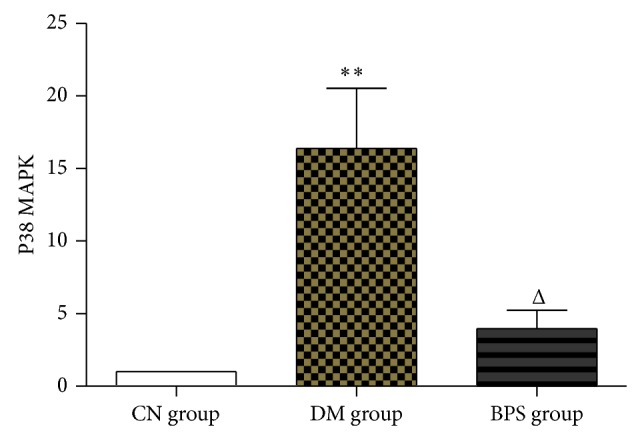
RT-PCR results of p38 MAPK gen. Compared with the CN group, the expression of p38 MAPK mRNA in the myocardial tissues of rats significantly increased in the DM group (*P* < 0.01) and also increased in the BPS group (*P* > 0.05). Compared with the DM group, the expression of p38 MAPK mRNA in rats in the BPS group significantly decreased (*P* < 0.01) and was similar to the level in the CN group (*P* > 0.05).

*RT-PCR Results of p38 MAPK Gene*. Compared with the CN group, the expression of p38 MAPK mRNA in the myocardial tissues of rats significantly increased in the DM group (*P* < 0.01) and also increased in the BPS group (*P* > 0.05). Compared with the DM group, the expression of p38 MAPK mRNA in rats in the BPS group significantly decreased (*P* < 0.01) and was similar to the level in the CN group (*P* > 0.05) (Figure 6).

**Figure 7:**
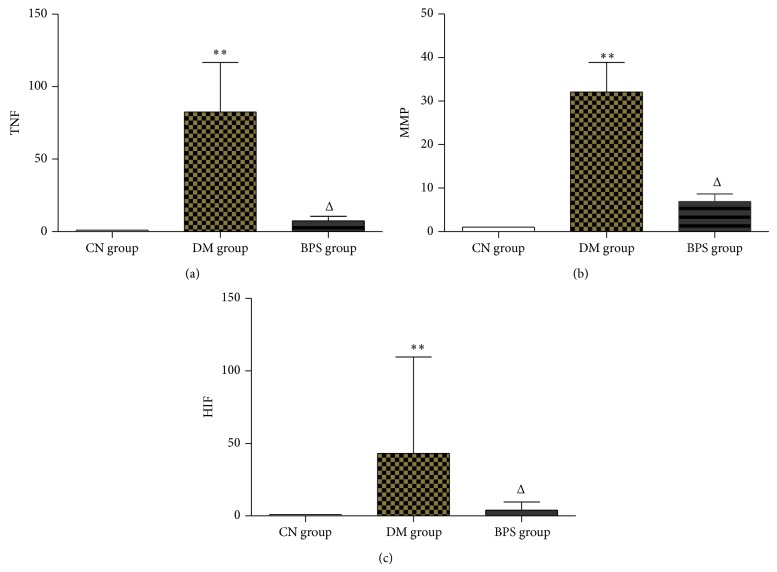
RT-PCR results of inflammatory factors. Compared with the CN group, the mRNA for TNF-*α*, MMP-9, and HIF-1*α* in the myocardial tissues of rats significantly increased in the DM group (*P* < 0.01) and also increased in the BPS group (*P* > 0.05). Compared with the DM group, the levels of TNF-*α*, MMP-9, and HIF-1*α* mRNA in rats of the BPS group significantly decreased (*P* < 0.01) and were similar to the levels or rats in the CN group (*P* > 0.05).

*RT-PCR Results of Inflammatory Factors*. Compared with the CN group, the mRNA for TNF-*α*, MMP-9, and HIF-1*α* in the myocardial tissues of rats significantly increased in the DM group (*P* < 0.01) and also increased in the BPS group (*P* > 0.05). Compared with the DM group, the levels of TNF-*α*, MMP-9, and HIF-1*α* mRNA in rats of the BPS group significantly decreased (*P* < 0.01) and were similar to the levels or rats in the CN group (*P* > 0.05) (Figure 7).

**Figure 8:**
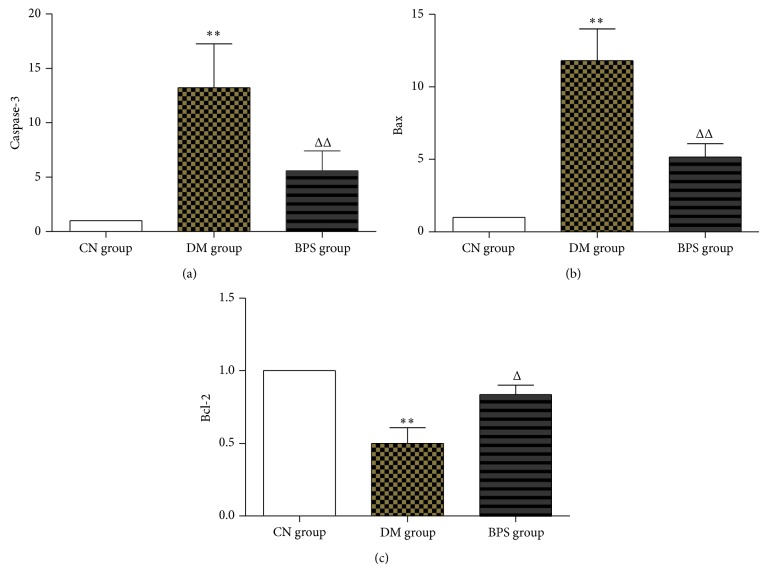
RT-PCR results of apoptosis genes in the myocardial tissues of each group. Compared with the CN group, the expression of caspase-3 and Bax mRNA significantly increased (*P* < 0.01) and the expression of Bcl-2 mRNA significantly decreased (*P* < 0.01) in the myocardial tissues of rats in the DM group. In the BPS rats, the expression of caspase-3 and Bax mRNA increased, and the expression of Bcl-2 mRNA decreased (*P* > 0.05). Compared with the DM group, the expression of caspase-3 and Bax mRNA significantly decreased and the expression of Bcl-2 mRNA significantly increased in BPS rats (*P* < 0.05).

*RT-PCR Results of Apoptosis Genes in the Myocardial Tissues of Each Group*. Compared with the CN group, the expression of caspase-3 and Bax mRNA significantly increased (*P* < 0.01) and the expression of Bcl-2 mRNA significantly decreased (*P* < 0.01) in the myocardial tissues of rats in the DM group. In the BPS rats, the expression of caspase-3 and Bax mRNA increased, and the expression of Bcl-2 mRNA decreased (*P* > 0.05). Compared with the DM group, the expression of caspase-3 and Bax mRNA significantly decreased and the expression of Bcl-2 mRNA significantly increased in BPS rats (*P* < 0.05) (Figure 8).

**Figure 9:**
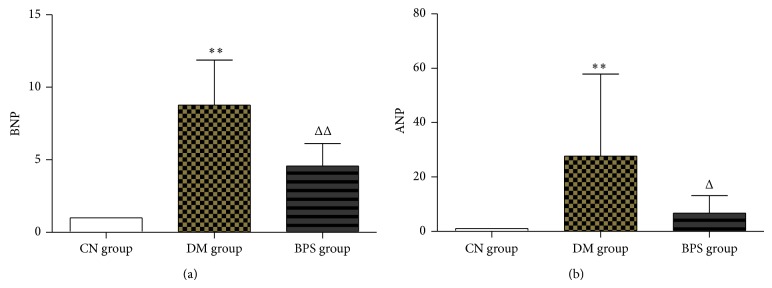
RT-PCR results of myocardia-associated hormone genes. Compared with the CN group, BNP and ANP mRNA in the myocardial tissues of rats in the DM group significantly increased (*P* < 0.01), whereas the expression of BNP and ANP mRNA in rats of the BPS group also increased (*P* > 0.05). Compared with the DM group, the expression of BNP and ANP mRNA in rats of the DM group significantly decreased (*P* < 0.05).

*RT-PCR Results of Myocardia-Associated Hormone Genes*. Compared with the CN group, BNP and ANP mRNA in the myocardial tissues of rats in the DM group significantly increased (*P* < 0.01), whereas the expression of BNP and ANP mRNA in rats of the BPS group also increased (*P* > 0.05). Compared with the DM group, the expression of BNP and ANP mRNA in rats of the DM group significantly decreased (*P* < 0.05) (Figure 9).

### 3.4. Western Blot Results

**Figure 10:**
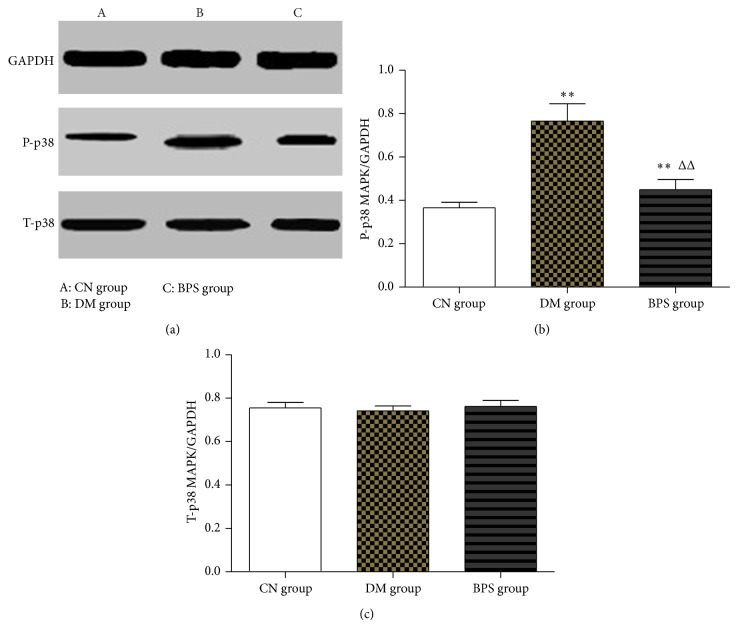
P-p38 MAPK and t-p38 MAPK protein levels in the myocardia. Compared with the CN group, the amount of p-p38 MAPK protein in the DM and BPS groups significantly increased (*P* < 0.01). Compared with the DM group, the amount of p-p38 MAPK protein in the BPS group significantly decreased (*P* < 0.01). Compared with the CN group, the amount of t-p38 MAPK protein in the DM and BPS groups did not significantly change (*P* > 0.05).

*P-p38 MAPK and t-p38 MAPK Protein Levels in the Myocardia*. Compared with the CN group, the amount of p-p38 MAPK protein in the DM and BPS groups significantly increased (*P* < 0.01). Compared with the DM group, the amount of p-p38 MAPK protein in the BPS group significantly decreased (*P* < 0.01). Compared with the CN group, the amount of t-p38 MAPK protein in the DM and BPS groups did not significantly change (*P* > 0.05) (Figure 10).

**Figure 11:**
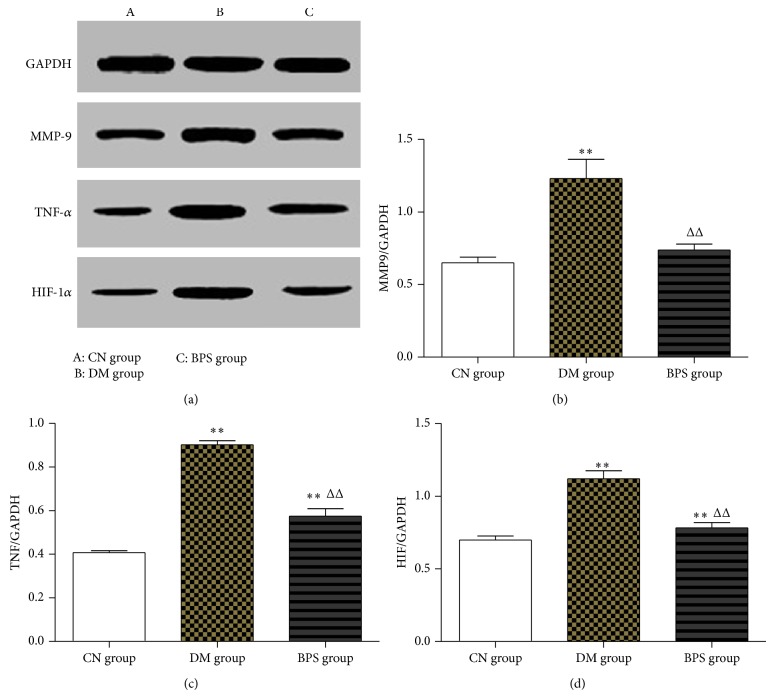
Protein expression levels of inflammatory factors in the myocardia. Compared with the CN group, MMP-9, TNF-*α*, and HIF-1*α* expression in the myocardial tissues of rats in the DM and BPS groups significantly increased (*P* < 0.01). Compared with the DM group, MMP-9, TNF-*α*, and HIF-1*α* expression in the BPS group significantly decreased (*P* < 0.01).

*Protein Expression Levels of Inflammatory Factors in the Myocardia*. Compared with the CN group, MMP-9, TNF-*α*, and HIF-1*α* expression in the myocardial tissues of rats in the DM and BPS groups significantly increased (*P* < 0.01). Compared with the DM group, MMP-9, TNF-*α*, and HIF-1*α* expression in the BPS group significantly decreased (*P* < 0.01) (Figure 11).

**Figure 12:**
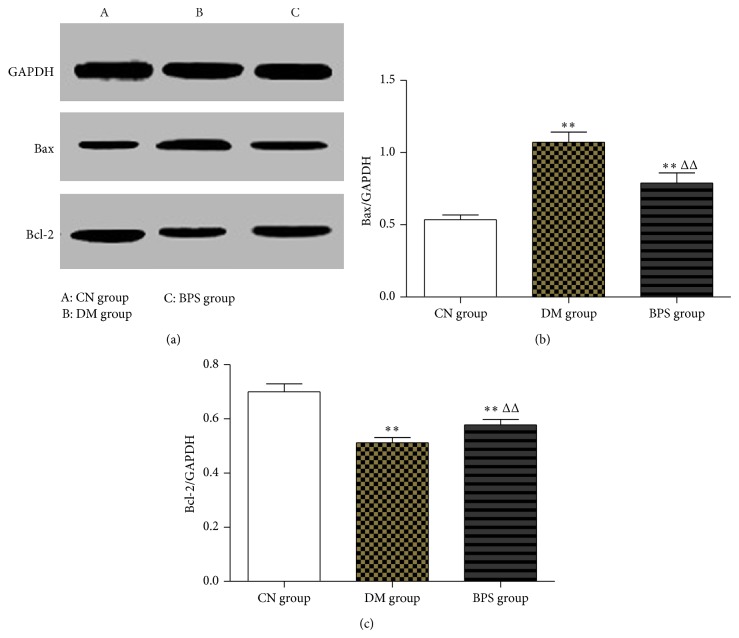
Protein level of apoptosis genes in the myocardia. Compared with the CN group, the expression of the Bax protein in the myocardial tissues of rats in the DM and BPS groups significantly increased (*P* < 0.01). Compared with the DM group, the expression of the Bax protein in the BPS group significantly decreased (*P* < 0.01). Compared with the CN group, the expression of the Bcl-2 protein in the myocardial tissues of rats in the DM and BPS groups significantly decreased (*P* < 0.01). Compared with the DM group, the expression of the Bcl-2 protein in the BPS group significantly increased (*P* < 0.01).

*Protein Level of Apoptosis Genes in the Myocardia*. Compared with the CN group, the expression of the Bax protein in the myocardial tissues of rats in the DM and BPS groups significantly increased (*P* < 0.01). Compared with the DM group, the expression of the Bax protein in the BPS group significantly decreased (*P* < 0.01). Compared with the CN group, the expression of the Bcl-2 protein in the myocardial tissues of rats in the DM and BPS groups significantly decreased (*P* < 0.01). Compared with the DM group, the expression of the Bcl-2 protein in the BPS group significantly increased (*P* < 0.01) (Figure 12).

**Figure 13:**
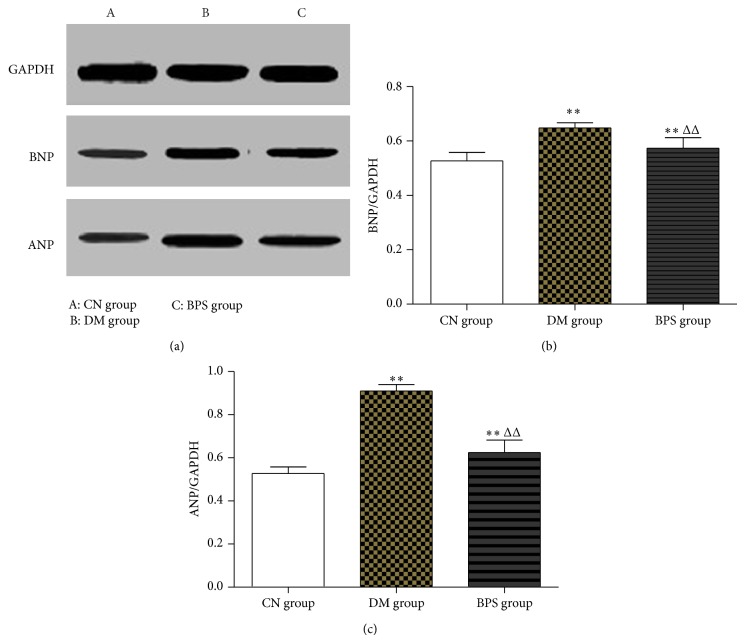
Expression levels of myocardia-associated hormone proteins. Compared with the CN group, the expression of the BNP and ANP proteins in the myocardial tissues of rats in the DM and BPS groups significantly increased (*P* < 0.01). Compared with the DM group, the expression of the BNP and ANP proteins in the BPS group significantly decreased (*P* < 0.01).

*Expression Levels of Myocardia-Associated Hormone Proteins*. Compared with the CN group, the expression of the BNP and ANP proteins in the myocardial tissues of rats in the DM and BPS groups significantly increased (*P* < 0.01). Compared with the DM group, the expression of the BNP and ANP proteins in the BPS group significantly decreased (*P* < 0.01) (Figure 13).

### 3.5. Pathological Findings of the Heart under Light Microscopy

**Figure 14:**
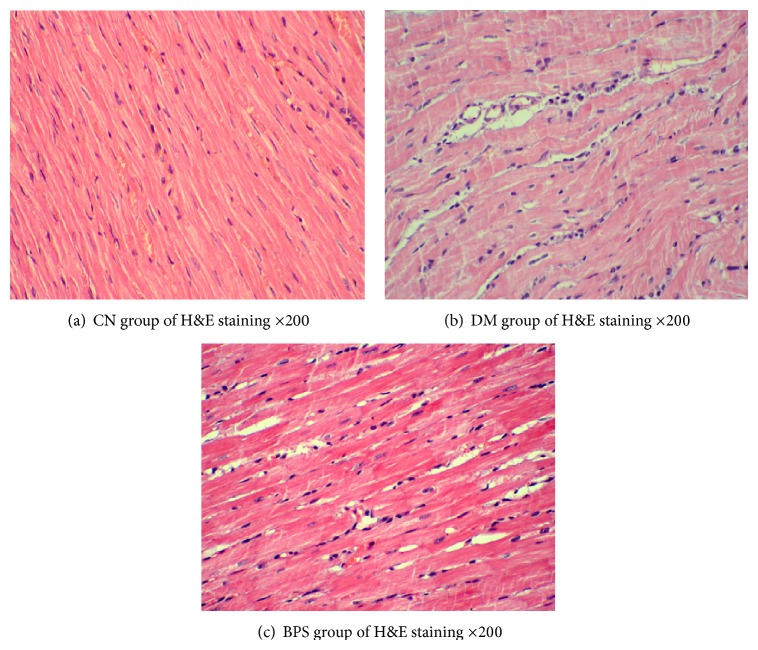
(a–c): H&E staining results. Cardiac structure of CN group was normal (a); H&E staining in DM group shows the cardiac muscle fibers derangement, the widening gap, the pyknotic and fragmented nuclei, and the increased apoptosis (b); after BPS treatment, the pathological damage significantly alleviated compared with the DM group (c).

The H&E staining of the myocardium of the NC group were presented as normal (Figure 14(a)), while the DM group showed irregular myocardial fiber structure, more scattered cells, nuclei of varying sizes, and more apoptotic nuclei (Figure 14(b)). Treatment with BPS markedly alleviates the pathological changes (Figure 14(c)).

### 3.6. Apoptosis of Myocardia

**Figure 15:**
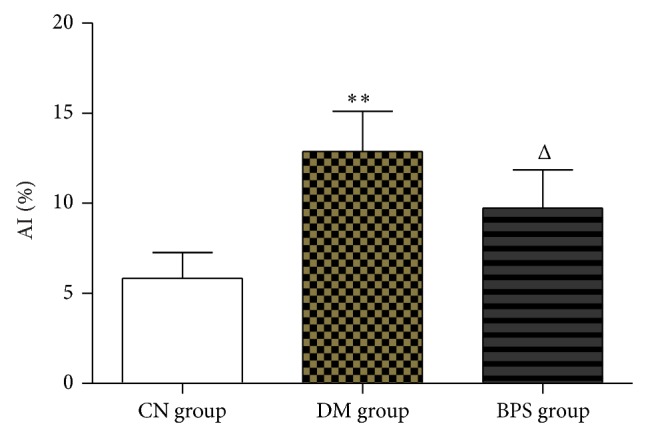
Apoptotic index. The DM group has the largest number of positive staining cells. The CN group has the minimum number of positive staining cells (*P* < 0.01); compared with the DM group, the number of positive staining cells in the BPS group was significantly decreased (*P* < 0.05).

**Figure 16:**
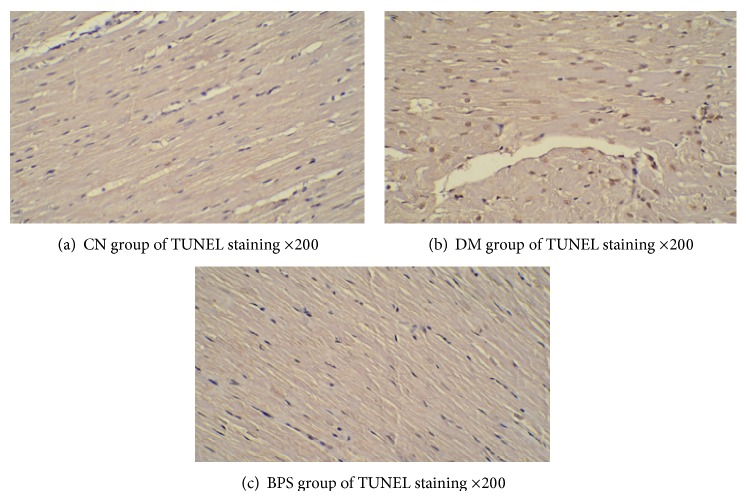
(a–c): Myocardial cell apoptosis results. Myocardial cell TUNEL staining is shown in (a)–(c). The CN group has the minimum number of positive staining cells (a). The DM group has the largest number of positive staining cells (b); compared with the DM group, the number of positive staining cells in the BPS group was significantly decreased (c).

TUNEL staining disclosed that apoptosis of the myocardia of the DM group is significantly exacerbated compared with that of the CN group (*P* < 0.01); compared with the DM group, the number of positive staining cells in the BPS group was significantly decreased (*P* < 0.05) (Figure 15). Myocardial cell TUNEL staining is shown in Figures 16(a)–16(c).

## 4. Discussion

This study showed that through the inhibition of the p38 MAPK signaling pathway activity, BPS reduced the expression of inflammatory factors such as TNF-*α*, HIF-1*α*, and MMP-9; inhibited myocardial cell apoptosis; and decreased the expression of BNP and ANP, thus delaying the progression of DCM and protecting cardiac function.

The MAPK family plays an important role in intracellular signal transduction and the onset and development of CVD; in particular, the p38 MAPK signaling pathway is closely associated with the onset and development of DCM [6, 15–18]. p38 MAPK uses a highly conserved three-kinase cascade to transduce signals. Extracellular stimuli activate and phosphorylate MKKK (MAP kinase kinase kinase), thus activating MKK (MAP kinase kinase). Next, the p38 MAPK signal transduction pathway is activated through the double phosphorylation of p38 MAPK into p-p38 MAPK, which participates in apoptosis, immune regulation, cellular transdifferentiation, and inflammatory reactions under oxidative stress [19].

The MAPK family includes extracellular signal-regulated kinases (ERK1, 2), c-Jun N-terminal kinases (JNK1, 2, 3), and p38 MAPK (*α*, *β*, *γ*, *δ*), which are all activated by oxidative stress and then activate downstream transcription factors including ATF-2, NF-*κ*B, and MEF-2, thus causing a series of inflammatory responses and apoptosis [20, 21]. Increasing evidence from animal and human studies confirmed a causal relationship between the p38 MAPK signaling pathway and DCM [20, 21]. The p38 MAPK signaling pathway can be activated by high glucose and DM. High glucose has adverse effects in different cell lines including vascular endothelial cells; the mechanisms of these effects involve advanced glycation end products, oxidative stress responses, abnormal sorbitol, and inositol metabolism and activation of diacylglycerol-protein kinase C (PKC). Further studies in aortic endothelial cells of STZ-induced diabetic rats showed that PKC activated the p38 MAPK signaling pathway in a high-glucose environment.

As a stress-activated kinase, p38 can be activated by chemical and physical factors, inflammatory factors, vasoactive and growth factors, cytokines such as TNF-*α*, ultraviolet rays, osmotic stimulation, oxidative stress, and microbial pattern recognition (Toll receptor adaptor), which is a common signal transduction pathway upstream of cell proliferation and differentiation, apoptosis, and necrosis. It is believed that p-p38 MAPK reflects the activity of p38 MAPK. Activated p38 MAPK is closely associated with cardiac damage [22]. Recent studies showed that the activation of the p38 MAPK signaling pathway causes overgrowth, proliferation, and differentiation of cells, which might be a common pathway for the onset and development of chronic complications of DM. The significantly increased levels of p-p38 MAPK in the myocardia of diabetic mice could be responsible for inflammation and the production of cytokines, with the consequence of significant damage to vascular endothelial cells and cardiac function. Recent studies showed that the use of p38 MAPK-specific inhibitors inhibited the activity of p-p38 MAPK and significantly increased cardiac function [23, 24]. However, thus far, most studies have used* in vitro* experiments; there were few* in vivo* experiments. In addition, specific p38 MAPK inhibitors are very expensive and are therefore difficult to use extensively in the clinic.

BPS has extensive functions in the prevention and treatment of microvascular complications of DM [25]. It was reported that BPS significantly reduced left ventricular end-diastolic pressure, ST/R ratio, and plasma creatinine kinase (CK) activity [26], which both prevented and treated DCM. A new study by Sato et al. [27] showed that BPS improved insulin resistance and abnormal glucose tolerance and reduced proteinuria in obese rats. Therefore, it was speculated that the therapeutic effect of BPS on microvascular complications of DM might be due to the improvement of glucose and lipid metabolism and a reduction of oxidative stress.

Our study showed that, compared with the CN group, the expression of p38 MAPK mRNA in the myocardial tissues of rats in the DM group significantly increased (*P* < 0.01) and that p38 MAPK also increased in the BPS group (*P* > 0.05). Compared with the DM group, the expression of p38 MAPK mRNA in the BPS group significantly decreased (*P* < 0.01). In addition, further detection of p-p38 protein level using Western blotting showed that the level of p-p38 in the DM group was significantly higher than that of the CN group. These results indicated that the p38 MAPK signaling pathway was significantly activated in myocardial tissues in diabetic rats, whereas the level of p-p38 was significantly lower than that of the DM group after BPS intervention. Compared with the CN group, t-p38 MAPK protein did not significantly change in the myocardial tissues of rats in the DM and BPS groups (*P* > 0.05). The results of this study suggested that, in the type-2 DM rat model, the p38 MAPK signaling pathway was activated, the production of related inflammatory factors increased, and cardiac injury accelerated. BPS inhibited the production of inflammatory factors and protected the heart by decreasing the activity of the pathway; these beneficial effects were closely associated with a decrease in inflammation and oxidative stress. These results indicated an important role for p38 MAPK in the stimulation of inflammatory signaling pathways and also showed a significant anti-inflammatory function of BPS.

Inflammation and progression of fibrosis induced by oxidative stress play important roles in the onset and development of DCM. The p38 MAPK signaling pathway links many cytokines and growth factors that can activate p38 MAPK to inflammation and oxidative stress injury, thus exacerbating cardiac injury in DM.

Our results demonstrated that the serum TGF-*β*1, MDA, TNF-*α*, MMP-9, and HIF-1a levels were significantly higher and SOD and GSH levels were markedly lower than that of the CN group (*P* < 0.01), showing that the oxidative stress was significantly enhanced and inflammatory cytokine production was increased. BPS treatment significantly decreased the serum MDA, TNF-*α*, MMP-9, and HIF-1a levels of DM group (*P* < 0.05).

TGF-*β*1 is one of the most important cytokines associated with myocardial fibrosis and one of the common mediators in the late stage of myocardial fibrosis. In cytology, TGF-*β*1 promotes the growth of fibroblasts, osteoblast, and Schwann cells. An abnormal increase of TGF-*β*1 plays a crucial role in the onset and development of myocardial fibrosis.

SOD is a generally accepted “free radical cleaner.” Serum SOD in type-2 DM patients is decreased, and the MDA level is increased [28], which makes the study of the role of SOD and free radical in DM possible. MDA promotes the crosslink between nucleic acid, protein, and lipid, resulting in mutation, degeneration, senescence, or even death of cells. The more serious the oxidative stress is, the greater the organism's antioxidant ability is and the greater the insulin resistance is [29]. SOD are enzymes that remove the toxic superoxide radicals* in vivo*, by catalyzing the chain reaction of lipid peroxidation (LPO), to protect the cells from damage. Therefore, an increase of SOD levels may achieve the balance between the production and reduction of free radicals and may be very important in the prevention and control of the chronic diabetic vasculopathy. GSH is a widely distributed peroxidase, catalyzing toxic peroxides into ordinary carbonyl compounds, reducing LPO caused by reactive oxygen species such as free radicals, preventing damage to important cellular components, and showing an antisenility effect to some extent.

TNF-*α* is one of these cytokine networks and is involved in many inflammatory responses, which can induce the release of many types of cytokines. During the induction of inflammatory responses, TNF-*α* has a chemotactic function on neutrophils and monocytes and can cause their activation and degranulation to release inflammatory mediators. It was confirmed that TNF-*α* induces the expression of adhesion molecules by vascular endothelial cells. Adhesion molecules attach to inflammatory cells and enhance the expression of procoagulant factors and plasminogen activator inhibitors in endothelial cells, thus promoting a series of effects such as intravascular thrombosis and the proliferation of endothelial and vascular smooth muscle cells. The heart is both the location of TNF-*α* production and the target organ of TNF-*α*. The overexpression of TNF-*α* is harmful to the heart. TNF-*α* inhibits the expression of glucose transporter type 4 in myocardial cells, decreases glucose utilization, depletes myocardial ATP, decreases ATP-dependent Na+-Ca2+ exchange on muscle fiber membranes, causes intracellular calcium overload, and affects myocardial systolic and diastolic function [30]. However, TNF-*α* also induces the overexpression of inducible nitric oxide synthase (iNOS) and the production of a large amount of NO, thus inhibiting normal myocardial contraction [31]. In addition, TNF-*α* also acts as a mediator of myocardial apoptosis [32]; pretreatment with TNF-*α* monoclonal antibodies significantly reduces myocardial apoptosis.

The activation of the p38 MAPK signaling pathway promotes the activation of inflammatory factors, promotes TNF-*α* synthesis, activates TNF-*α*-mediated E-selectin expression, and regulates the expression of TNF-*α*-induced vascular cell adhesion molecule 1 (VCAM-1) in epithelial cells. Of the MAPK family members, ASK1 activates two different kinases: MAPKK-SEK1 (MKK4) and MKK3/MAPKK6 (MKK6). The latter activates the p38 MAPK signaling pathway. Studies showed that ASK1 was activated by the action of TNF-*α* [33], indicating that TNF-*α* in turn activates the p38 MAPK signaling pathway through the activation of MAPKKK upstream of p38 MAPK [34]. This study determined that TNF-*α* expression in rats of the DM group significantly increased compared with that of rats in the CN group (*P* < 0.01), suggesting that there was a significant inflammatory response in the hearts of rats in the DM group. The expression levels of TNF-*α* significantly decreased after BPS intervention (*P* < 0.01). The results of this study suggested that BPS decreased the levels of TNF-*α* expression, inhibited p38 MAPK activity, and reduced inflammatory responses in the heart.

The MMP family is a group of highly conserved, zinc-dependent endopeptidases that use extracellular matrix components as hydrolysis substrates. MMPs function in many pathophysiological processes including inflammatory reactions, embryonic development, immune responses, tissues remodeling, and tumor metastasis [35]. MMP-9, a member of this family also known as gelatinase, primarily hydrolyzes denatured collagen and is closely associated with CVD. Recent studies showed that many cytokines such as TNF-*α* upregulate MMP-9 expression. MMP-9 is the end product of inflammation and acts as an inflammatory mediator that participates in inflammatory reactions and tissue destruction. The activation of the p38 MAPK signaling pathway induces the production and increases the release of MMP-9. We also observed that MMP-9 expression in the type-2 DM rat model significantly increased compared with that of the control group (*P* < 0.01); after BPS intervention, the expression level of MMP-9 significantly decreased in the intervention group (*P* < 0.01). These results suggest that MMP-9 is involved in the development of inflammation during the progression of DCM. Through the inhibition of p38 activity, BPS could decrease the expression level of MMP-9 in the heart and delay inflammatory injury.

HIF-1*α* is a nuclear transcriptional regulator that participates in the onset and development of DCM through the regulation of its downstream genes. Under hypoxia, HIF-1*α* promotes angiogenesis by activating vascular endothelial growth factor (VEGF); thus, the metabolism of the body can be adapted to hypoxic environments. The effect of HIF-1*α* on increasing the levels of VEGF is decreased in DM patients [36]. HIF-1*α* is present in the cytoplasm and nucleus of myocardial cells. The increase in HIF-1*α* expression in the DM group was significantly higher than that in the control group, indicating that the increased HIF-1*α* expression in DM promoted myocardial apoptosis. p38 MAPK activates HIF-1*α* in vascular smooth muscle cells and regulates the expression of HIF-1*α* through the phosphorylation of HIF-1*α*. Our study observed that HIF-1*α* expression in the type-2 DM rat model was significantly higher than that in the control group (*P* < 0.01); after BPS intervention, HIF-1*α* expression significantly decreased in the intervention group (*P* < 0.01). The results of this study suggested that, by inhibiting p38 activity, BPS decreased the expression level of HIF-1*α* in the heart and delayed heart failure.

Apoptosis is one important cause of cardiac insufficiency in DCM. Apoptosis of myocardial cells participates in the pathological process of many CVDs, including cardiomyopathy, myocardial infarction, and congestive heart failure. The caspase family is a group of proteases containing caspase-3. As a common “central processor” of apoptosis pathways, caspases not only mediate B cell apoptosis and participate in the onset and development of DM but also mediate myocardial cell apoptosis. Myocardial cell apoptosis may be one of the causes of the loss of myocardial cells and heart failure in DCM. Caspase-3 is a “core” protease in the Fas-mediated caspase cascade. Fas-mediated apoptosis also involves p38 MAPK; the activation of the p38 MAPK pathway activates caspase-3 and begins the apoptosis process. Our study observed that the expression of caspase-3 in the type-2 DM rat model was significantly higher than that in the control group (*P* < 0.01), whereas after BPS intervention, the level of caspase-3 in the intervention group significantly decreased (*P* < 0.05). These results suggested that BPS reduces the level of caspase-3 in the heart, decreases myocardial apoptosis, and protects cardiac function by inhibiting p38 activities.

Bcl-2 and Bax both are important apoptosis-related genes. Bcl-2 is a mitochondrial inner membrane protein that inhibits apoptosis. The main functions of Bcl-2 are to promote cell survival, prolong cell lifespan, and inhibit apoptosis. In contrast to Bcl-2, Bax promotes apoptosis, although Bax is also a Bcl-2 family member. The relative concentrations and balance between these two proteins play important roles in the regulation of apoptosis. Bcl-2 family proteins act upstream of mitochondria; these proteins regulate the permeability of the mitochondrial membrane, thus regulating the activation of downstream caspase proteases and mediating cell survival or death [37]. It was reported that Bcl-2/Bax were important components for MAPKs to exert their functions [38–40]. Bcl-2 forms a dimer with the membrane-bound ligand Bax. When Bcl-2 is in excess, the formation of Bcl-2/Bax heterodimers can prevent apoptosis; when Bax is in excess, the formation of Bax/Bax homodimers promotes apoptosis. Therefore, the ratio of Bcl-2/Bax determines the sensitivity of cells to apoptosis-inducing signals. Under normal circumstances, p38 MAPK is located in the cytoplasm; once activated, it will be rapidly translocated into the nucleus to activate MAPK-activated protein kinases 2 and 3 and caspase family members. In our study, we observed that the expression of Bcl-2 mRNA in the type-2 DM rats was significantly lower than that in the control group (*P* < 0.01), whereas the expression of Bax mRNA was significantly higher than that in the control group (*P* < 0.01). The ratio of Bcl-2/Bax significantly decreased, indicating that myocardial apoptosis in DCM increased. After BPS intervention, the Bcl-2 level significantly increased in the intervention group and the Bax level significantly decreased (*P* < 0.05). These results suggested that BPS decreased myocardial apoptosis and protected myocardial cells of DCM dependent on the Bcl-2/Bax ratio.

Some peptide neurohormones play important roles in the determination of diagnosis and treatment of DCM; in addition, their concentrations are closely associated with the prognosis of DCM. BNP is a hormone which is secreted by ventricular myocytes; myocardial ischemia, necrosis and injury, ventricular wall tension, and high pressure stimulate the synthesis and secretion of BNP. BNP is then released into the peripheral blood, which significantly increases the concentration of BNP in the blood of patients [41]. Although the concentration of BNP reflects the degree of myocardial ischemia and necrosis, it also positively correlates with the severity of heart failure and occurs before myocardial necrosis. A persistent increase in BNP value is an independent risk factor of death from heart failure [42]. Phosphorylation of p38 MAPK also accelerates the process of myocardial hypertrophy [43]. In our study, we observed that BNP expression in the type-2 DM rat model was significantly higher than that in the control group (*P* < 0.01), suggesting that there was significant myocardial ischemia and necrosis in DM rats. After BPS intervention, the BNP level significantly decreased in the intervention group (*P* < 0.05). The results showed that BPS decreased the BNP level in the heart and indicated that BPS alleviated myocardial ischemia, delayed heart failure, and protected cardiac function.

ANP is an endocrine hormone mainly synthesized and secreted by cardiac tissues (mainly the atria) that is closely associated with cardiac function. An increase in left atrial pressure and volume load can stimulate the atrial wall pressure-volume receptor, thus increasing ANP secretion by myocardial cells [44]. DCM typically causes myocardial hypertrophy and stretching, which may cause changes in ANP levels. Thus far, much experimental and clinical evidence has shown that because the plasma ANP level is directly associated with left ventricular pressure, at the early stage of some cardiac diseases, the circulating level of ANP reflects the early stage of cardiac dysfunction, or in other words, the ANP level reflects the presence and severity of asymptomatic left ventricular dysfunction [45]. With the aggravation of myocardial hypertrophy, a continuous increase in ANP secretion by ventricular myocytes significantly correlates with the degree of ventricular hypertrophy. Our studies showed that the expression of ANP in the type-2 DM rat model was significantly higher than that in the control group (*P* < 0.01), suggesting that there were significant ventricular hypertrophy and increased left ventricular pressure in DM rats. After BPS intervention, the ANP level in the intervention group significantly decreased (*P* < 0.05). These results showed that BPS decreased ANP levels in the heart and indicated that BPS reduced ventricular hypertrophy and improved left ventricular dysfunction.

However, due to the limitations of experimental conditions, this study did not involve cardiac Doppler examination in diabetic rats to further clarify the condition of cardiac functions. The relationship between different types of p38 MAPK and DCM and the specific target of BPS also requires further studies.

Overall, our animal studies showed that by activating the p38 MAPK signaling pathway, BPS inhibited the production of inflammatory factors caused by oxidative stress in type-2 DM, decreased the protein levels of HIF-1*α*, TNF-*α*, and MMP-9 in myocardial tissues, downregulated caspase-3 levels, and increased the ratio of Bcl-2/Bax, thus decreasing inflammatory injury, reducing myocardial apoptosis, improving myocardial ischemia and myocardial hypertrophy, delaying heart failure, and delaying the progression of DCM. This study confirmed the protective effect of BPS on the heart and its possible underlying mechanism using animal experiments; these data provide new ideas for the clinical treatment of DCM.

## Figures and Tables

**Table 1:** Primer sequences.

Primer	Sequence (5′-3′)	Annealing temperature (°C)
p38 MAPK	Forward primer: 5′-TTCCCAGCAGTCCTATCC-3′	55
	Reverse primer: 5′-GTCAGATGGCAAGGGTTC-3′	

GAPDH	Forward primer: 5′-TTGCTGATGACTGGTTACAATACA-3′	55
	Reverse primer: 5′-GCTTGACTTACAGAAGAATCGTTG-3′	

## Abbreviations

TNF-*α*: Tumor necrosis factor-*α*
HIF-1*α*: Hypoxia inducible factor 1*α* subunit
MMP-9: Matrix metalloproteinase-9
BNP: Brain natriuretic peptide
ANP: Atrial natriuretic peptide
DCM: Diabetic cardiomyopathy.
